# The Therapeutic Value of Relaxing Climates

**Published:** 1905-03-11

**Authors:** 


### THE THERAPEUTIC VALUE OF RELAXING CLIMATES.

In an article on this subject Dr. Leonard Williams[1] argues that climatotherapy and the principles which underlie it are much neglected both in the medical schools and in medical practice. Change of air is doubtless a frequent prescription, and in so far as it secures a pure atmosphere is to the good of the invalid and convalescent. But there is a widespread failure to recognise that other elements than purity of atmosphere have to be considered before the therapeutic value of any particular climate can be appreciated. Such factors as moisture, sunshine, and exposure all take their share in influencing metabolic changes, and hence ought to be considered in the effort to guide the application of climatic treatment in any individual case. A particular illustration of this want of information and judgment may be found in the undiscriminating fashion in which "a bracing climate" is advised. Refreshing and stimulating as such a climate is to persons in moderately good health, it may exercise a disastrous influence in certain pathological conditions. On the other hand, a "relaxing climate," though possessed of few attractions for the healthy and apt thus to come under a general condemnation, is possessed of a much wider therapeutic range than one which is more grateful to the vigorous and robust. Its action is to decrease metabolism and to lower generally the activity of the vital processes. But it is this sedative tendency rather than the stimulating effect of a bracing climate that is needed in most chronic invalids, and particularly in sufferers from chronic pulmonary or renal troubles as well as in cases of chronic heart disease and in chronic nervous disease, this general statement being qualified by the principle that in climatic, as in all other forms of treatment, the peculiarities of the individual patient require exact study. In pulmonary affections an example of the value of a relaxing climate may be found in the treatment of emphysema. The moisture of the atmosphere in such a climate acts as a sedative to the respiratory passages and, by minimising changes in temperature protects the patient from the extremes to which, in a bracing climate, he would probably be exposed. Similarly in chronic renal disease the relaxing climate both diminishes the amount of waste material produced in the tissues and assists the action of the skin and lungs in their compensatory excretory function. Hence the work of the damaged kidneys is diminished and the dangerous complications which are associated with renal disease are delayed. Again, in chronic cardiac disease the lowered function and diminished blood-pressure which attend on life in a relaxing climate relieve the strain on the cardiac muscle, and so favour its possibilities of endurance. In the treatment of the degenerative processes which affect the central nervous system the relaxing climate is the only safe medium. The vital forces have to be conserved so that the unfavourable movement of the morbid process may be checked. In neurasthenia and hysteria the therapeutic problem, so far as climate is concerned, is less simple. Temperamental considerations and local circumstances must be borne in mind, as also the mental attitude and reaction of the patient. The possibilities or otherwise in reference to suitable baths and other mechanical methods of treatment are also guiding influences in the selection of a suitable climatic station, and this more particularly in cases of chronic renal and chronic cardiac disease.

[1] Edin. Med. Jour., March 1905.

